# Participation of the nucleus tractus solitarius in the therapeutic effect of electroacupuncture on post‐stroke dysphagia through the primary motor cortex

**DOI:** 10.1111/cns.14442

**Published:** 2023-09-04

**Authors:** Qiuping Ye, Si Yuan, Lulu Yao, Yong Dai, Bing Deng, Jiahui Hu, Jiao Qiao, Hongmei Wen, Zulin Dou, Nenggui Xu

**Affiliations:** ^1^ Department of Rehabilitation Medicine The Third Affiliated Hospital of Sun Yat‐sen University Tianhe District, Guangzhou China; ^2^ Clinical Medical College of Acupuncture Moxibustion and Rehabilitation Guangzhou University of Traditional Chinese Medicine Panyu District, Guangzhou China; ^3^ Department of Rehabilitation of Traditional Chinese Medicine Hunan University of Chinese Medicine Yuelu District, Changsha China; ^4^ South China Research Center for Acupuncture and Moxibustion Guangzhou University of Traditional Chinese Medicine Panyu District, Guangzhou China

**Keywords:** electroacupuncture, nucleus tractus solitarius, post‐stroke dysphagia, primary motor cortex

## Abstract

**Background:**

Post‐stroke dysphagia (PSD), a common and serious disease, affects the quality of life of many patients and their families. Electroacupuncture (EA) has been commonly used effectively in the treatment of PSD, but the therapeutic mechanism is still under exploration at present. We aim to investigate the effect of the nucleus tractus solitarus (NTS) on the treatment of PSD by EA at Lianquan (CV23) through the primary motor cortex (M1).

**Methods:**

C57 male mice were used to construct a PSD mouse model using photothrombotic technique, and the swallowing function was evaluated by electromyography (EMG) recording. C‐Fos‐positive neurons and types of neurons in the NTS were detected by immunofluorescence. Optogenetics and chemical genetics were used to regulate the NTS, and the firing rate of neurons was recorded via multichannel recording.

**Results:**

The results showed that most of the activated neurons in the NTS were excitatory neurons, and multichannel recording indicated that the activity levels of both pyramidal neurons and interneurons in the NTS were regulated by M1. This process was involved in the EA treatment. Furthermore, while chemogenetic inhibition of the NTS reduced the EMG signal associated with the swallowing response induced by activation of M1 in PSD mice, EA rescued this signal.

**Conclusion:**

Overall, the NTS was shown to participate in the regulation of PSD by EA at CV23 through M1.

## Graphical abstract

Excitatory neurons of nucleus tractus solitarius participate in the treatment of dysphagia after stroke by electroacupuncture at Lianquan acupoint through primary motor cortex.

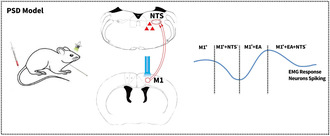

## 1 INTRODUCTION

Physiological swallowing is fundamental for human daily life and requires the coordination of complex nervous and muscular systems.^1^, ^2^ Previous studies have shown that the cerebral cortex plays an important role in swallowing, including the primary motor cortex (M1).^3^, ^4^ Regarding the subcortex areas, central pattern generators (CPGs) of swallowing include the nucleus tractus solitarus (NTS), nucleus ambiguus (NA), and ventrolateral medulla oblongata,^5^ which are believed to be involved in the swallowing reflex.^6^ CPG could be activated both by the peripheral nerve or the cortex, especially the primary sensorimotor cortex,^7^ the cortical fibers of which mainly project to the NTS.^8^ Therefore, the NTS can integrate and regulate information from both the higher cortex center and peripheral sensory afferents.^9^

Dysphagia is a common disease induced by neurological diseases, such as stroke, Parkinson's disease, Alzheimer's disease, and dementia, among which stroke is the most common cause of dysphagia.^10^, ^11^, ^12^, ^13^ Statistically, the probability of causing post‐stroke dysphagia (PSD) has reached 78%,^10^ and it has become a major disease that affects people's health and quality of life. PSD leads to numerous complications, including malnutrition, dehydration, and pneumonia due to aspiration, which might result in a poor survival outcome and increased mortality.^14^ For the treatment of PSD, except for rehabilitation methods, pharyngeal electrical stimulation (PES), repetitive transcranial magnetic stimulation (rTMS), neuromuscular electrical stimulation (NMES), transcranial direct current stimulation (tDCS), and acupuncture are commonly used clinically.^15^, ^16^, ^17^, ^18^ Among these treatment methods, acupuncture has been proven as an effective treatment for PSD in clinical studies.^19^, ^20^ Regarding its convenience, simple operation, and high acceptance in clinical practice, acupuncture has been widely used for analgesia,^21^ nausea, and vomiting.^22^

Electroacupuncture (EA), a complementary and alternative therapy for dysphagia after stroke, has gradually become accepted throughout the world and is also a commonly used method for the treatment of multiple diseases.^23^ CV23, located in front of the hyoid, is a common acupoint used for dysphagia,^24^ but its mechanism is still being explored. Our recently published article demonstrated that optogenetic activation of pyramidal neurons in layer 5 (L5) of M1 could induce swallowing responses.^25^ The therapeutic mechanism of EA at CV23 in PSD model mice was related to the activation of M1 and its inputs to the NTS through the parabrachial nuclei (PBN), providing a scientific basis for the action of M1 via the subcortical pathway in swallowing function. This study indicated that the NTS played an indirect role in the treatment of PSD by EA through the regulation of M1. M1 is believed to play an important role in voluntary swallowing, as has been demonstrated in many studies,^5^, ^26^ while the NTS is a subcortical area and plays an important role in the function of reflex swallowing.^5^ A previous study also showed that EA at CV23 could regulate neuronal activity in M1 and promote the recovery of dysphagia after stroke.^27^ However, it is not clear whether the NTS is directly involved in the therapeutic effect of EA at CV23 on swallowing function in PSD model mice through M1.

Therefore, we explored swallowing function in the context of optogenetic activation of the motor cortex‐induced swallowing process and the important role of the NTS during the process of regulation of PSD by EA through M1.

## 2 METHODS

### 2.1 Animals

C57BL/6J and GAD67‐GFP mice were purchased from the Laboratory Animal Center of Guangzhou University of Chinese Medicine. Mice aged 5–6 weeks (approximately 20–30 g), all male, were housed under a 12 h day/night cycle with free access to food and water. Homologous GAD67‐GFP mice were mated and identified before the experiment. Mice implanted with optical fibers were individually housed. All experimental procedures were performed following the guidelines of the Committee for Care and Use of Research Animals of Guangzhou University of Chinese Medicine (No. 201703303). For all experiments, mice were randomly assigned to the experimental and control groups.

### 2.2 Virus injection

AAV2/9 viruses were used and injected into C57 mice throughout the experiment, including rAAV2/9‐CaMKIIα‐hM4D(Gi)‐mCherry‐WPRE‐pA, rAAV2/9‐hSyn‐hM3D(Gq)‐EGFP‐WPRE‐pA, rAAV2/9‐CaMKIIα‐hChR2(E123T/T159C)‐mCherry‐WPRE‐hGH‐pA, rAAV2/9‐CaMKIIα‐EGFP‐WPRE‐pA, and AAV2/9‐CaMKIIα‐mCherry‐WPRE‐pA (BrainVTA, Wuhan). All viruses were injected into C57 mice. rAAV2/9‐CaMKIIα‐hChR2 (E123T/T159C)‐mCherry‐WPRE‐hGH‐pA were used and injected into M1 of GAD67‐GFP mice only in the neuron identification experiment in Figure 2. Mice were anesthetized with 1.25% tribromoethanol and fixed in a prone position in the adapter. The skull hair was cut off, and a line was cut along the midline of the scalp to expose the skull. Then, the tissue was removed. The target area was selected, and a hole was drilled. After opening the dura, a needle fixed to a stereotaxic instrument was filled with the target virus and slowly injected into the target area at a rate of 30 nL/min. After 10 min, the needle was slowly lifted, and mice were fed in the cage for 21 days before the experiment. In the optogenetic mice, an optical fiber was implanted above the virus injection site and fixed with dental cement.

### 2.3 PSD modeling

The photothrombotic technique was used to generate a PSD mouse model.^25^ Mice were anesthetized with isoflurane and injected with 15 mg/mL Rose Bengal solution. A 532‐nm wavelength laser beam was used to illuminate the target area for 8 min. Other regions were covered with shading paper to avoid the laser illumination. Afterward, mice were sutured and stayed on the electric blanket to recover from the anesthesia before being returned to their home cage. For more details of the method, please refer to our previous article.^25^

### 2.4 Electroacupuncture (EA)

To determine the effects of EA stimulation on the PSD mouse model, mice received EA stimulation 24 h after M1 ischemia surgery. Mice were anesthetized with 2% isoflurane in an oxygen/air mixture. A 0.16 × 10 mm unipolar stainless‐steel needle (Suzhou Medical Appliance Factory) was inserted obliquely (toward the root of the tongue) into CV23 at a depth of approximately 0.5 cm. Han's Acupoint and Nerve Stimulator (HANS, China) was used for intermittent pulse electrical stimulation with intensities of 2 Hz and 1 mA for 15 min.

### 2.5 Optogenetic activation

An AAV2/9 virus with the CaMKIIα promoter was used, which could specifically infect excitatory neurons to transfect M1 neurons through stereotaxic injection. An optical fiber was implanted 2.5 mm above the location of virus injection. Injection of a virus with a ChR2 photosensitive channel into M1 can open the cationic channels and induce the action potential when blue light is given. After adequate expression of the virus, mice were anesthetized, and an optical fiber (NEWDOON) was connected to the fiber on the head. Light was given at 473 nm and 50 Hz for 5 s using a stimulus modulation generator (Tinker Tech). During the c‐Fos staining experiment, mice were sampled after 50 min of light exposure, and the CaMKIIα promoter virus was injected into the NTS simultaneously. For the experiment of EMG recording, light was provided during the EMG recording.

### 2.6 Immunofluorescence

Three weeks after injection and modeling, mice were transcardially perfused and the brain was extracted, followed by postfixation with 4% paraformaldehyde (PFA). After gradual dehydration in 15% and 30% sucrose in PBS, brain tissue was sectioned coronally at a thickness of 40 μm with a freezing microtome (Thermo). Every third section of the NTS was left, and three pieces were taken from each mouse for the next experiment. After free‐floating washing in PBS, the brain slices were incubated for 1 h in blocking solution (1% BSA and 0.3% Triton X‐100 in PBS), followed by incubation with primary antibodies overnight at 4°C (c‐Fos, Cell Signaling Technology, catalog no. 2250; diluted 1:500) in blocking solution. The brain slices were then washed with PBS and incubated with secondary antibodies in 0.3% Triton X‐100 in PBS for 2 h at room temperature (conjugated to Alexa Fluor 488, catalog no. 711–545‐152; diluted 1:500). Finally, they were incubated with 4,6‐diamidino‐2‐phenylindole (DAPI; 1 μg/5 mL) after washing and mounted on glass slides. Afterward, confocal fluorescence images were obtained using 40X air objectives on a Nikon scanning laser microscope. Image analysis was performed using ImageJ or NIS‐Viewer v.4.5 (Nikon, Japan).

The expression of c‐Fos was manually quantified in the NTS by an observer (Si Yuan) blinded to the experimental conditions, with the guidance of a mouse brain atlas. The area was measured using ImageJ software (1.52a, National Institutes of Health, America). Next, the area of the NTS was measured on all images based on c‐Fos labeling. The c‐Fos‐positive neurons were calculated for each slice by dividing the total number of sampled cells by the total areas of the region. c‐Fos‐positive neurons were counted in a blinded manner in more than three sections from each mouse, and then the final values were averaged to obtain one value for each animal.

### 2.7 Electromyography (EMG) recording combined with optogenetics and chemogenetics

Swallowing response was detected by EMG recording combined with an optogenetic approach. EMG‐recording was performed 24 h after PSD modeling or EA stimulation. The mice were placed in a supine position and fixed on a homemade foam board. Two EMG recording electrodes were inserted into the pharyngeal muscle to record the EMG of the swallowing response. A ground electrode was inserted into one side of the masseter muscle. They were connected to the recording wires, which were linked to a signal acquisition system to acquire EMG signals (1902, CED). After recording the EMG during light administration, CNO (3 mg/kg, Sigma) was administered intraperitoneally to inhibit NTS neurons, and light was administered again. EMG was recorded throughout the experiment and was collected and analyzed with the Spike2 system (Spike2 1401, CED).

### 2.8 Balloon pressure detection

To further detect the swallowing response, a homemade balloon was used to detect the swallowing response. First, the balloon was fixed over the pharynx of mice in a supine position. The machine (Powerlab, ADInstruments) connected to the balloon was used to measure the pressure of the balloon during swallowing induced by optogenetic activation of M1. The pressure of the balloon were detected when light was given.

### 2.9 In vivo multichannel electrophysiological recording combined with chemogenetics

To record the firing rates of NTS neurons, 0.25 μL AAV2/9‐CaMKIIα‐hM3Dq‐mCherry, AAV2/9‐CaMKIIα‐hM4Di‐mCherry, or AAV2/9‐CaMKIIα‐EGFP was injected into the M1 L5 of mice. Fourteen days later, mice were anesthetized with isoflurane and fixed in a stereotaxic instrument (Desktop Digital stereoLocator, RWD, China). A seam was made in the middle of the scalp, and the skull was exposed. The target area was located, and a small hole was drilled by the skull drill. A multichannel electrode was implanted into the NTS, and an annular tube was secured with dental cement to facilitate modeling. The mice were then fed separately until they were recorded 7 days later. The plexon system was used for recording and analyses (OmniPlex in vivo multichannel recording system; NeuroExplorer 4.0; Offline Sorter 4.0, Plexon). Neurons were recorded for 5 min as baseline before CNO intraperitoneal injection. Thirty minutes after CNO injection, neuronal firing was recorded for 5 min again.

### 2.10 Statistical analysis

Normally distributed data were analyzed using one‐way ANOVA with Tukey's post hoc test (for the analysis of c‐Fos‐positive neurons, EMG recording, pharyngeal pressure, and NTS neurons) or an unpaired *t* test (for the analysis of neuronal types in NTS). *p* < 0.05 was considered a significant difference. The two‐tailed *F* values or *p* values are given in the Figure Legends. Data analysis was performed by an experimenter who was blinded to the experimental conditions. All statistical analyses were performed using GraphPad Prism 8.0 software. All the figures were made with sigmaplot 14. Data are presented as the mean ± SD, and *n* indicates the number of biologically independent samples (mice) per group.

## 3 RESULTS

### 3.1 Excitatory neurons in the NTS are mainly activated by M1

**FIGURE 1:**
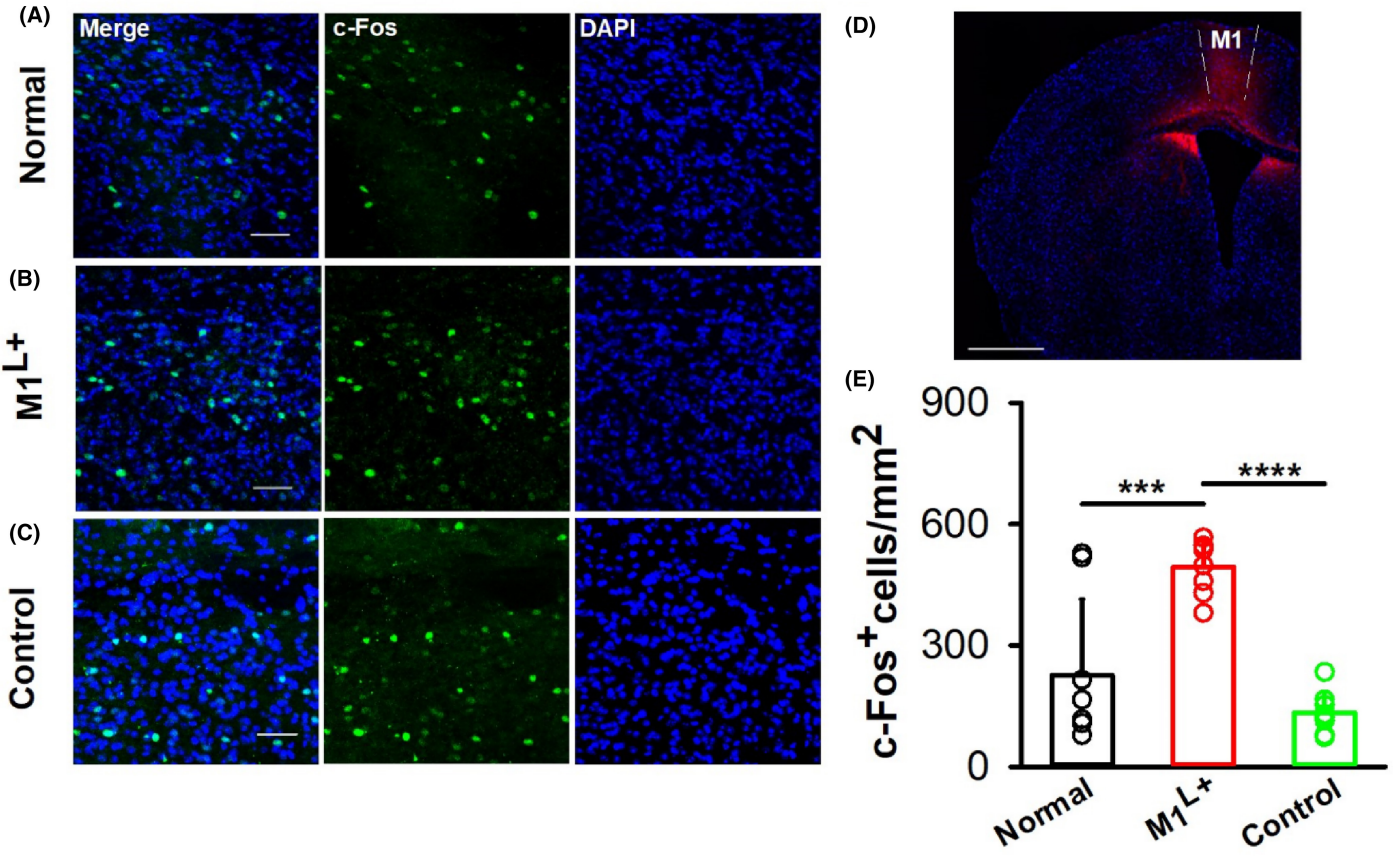
Excitatory neurons in M1 could activate c‐Fos‐positive neurons in the NTS. (A–C) Representative images of c‐Fos‐positive neurons in the NTS after activation by optogenetic methods in the normal (A), M1^L+^ groups (B) (AAV2/9‐CaMKIIα‐ChR2 virus was injected into M1, and blue light was delivered) and control group (C) (AAV2/9‐CaMKIIα‐EGFP virus was injected into M1, and blue light was delivered) of C57 mice. Scale bar, 50 μm. (D) The location of AAV2/9‐CaMKIIα‐ChR2‐mCherry virus injection in the M1. Scale bar: 500 μm. (E) The c‐Fos‐positive neurons of the NTS increased after optogenetic activation of M1 in the M1^L+^ group (one‐way ANOVA, *n* = 8 per group; *F* = 19.79, ***p < 0.001, normal vs. M1^L+^; ****p < 0.0001, M1^L+^ vs. control). L^+^ indicates that blue light was delivered to the M1. Data are presented as the mean ± SD and indicate the number of biologically independent samples (mice) per group.

**FIGURE 2:**
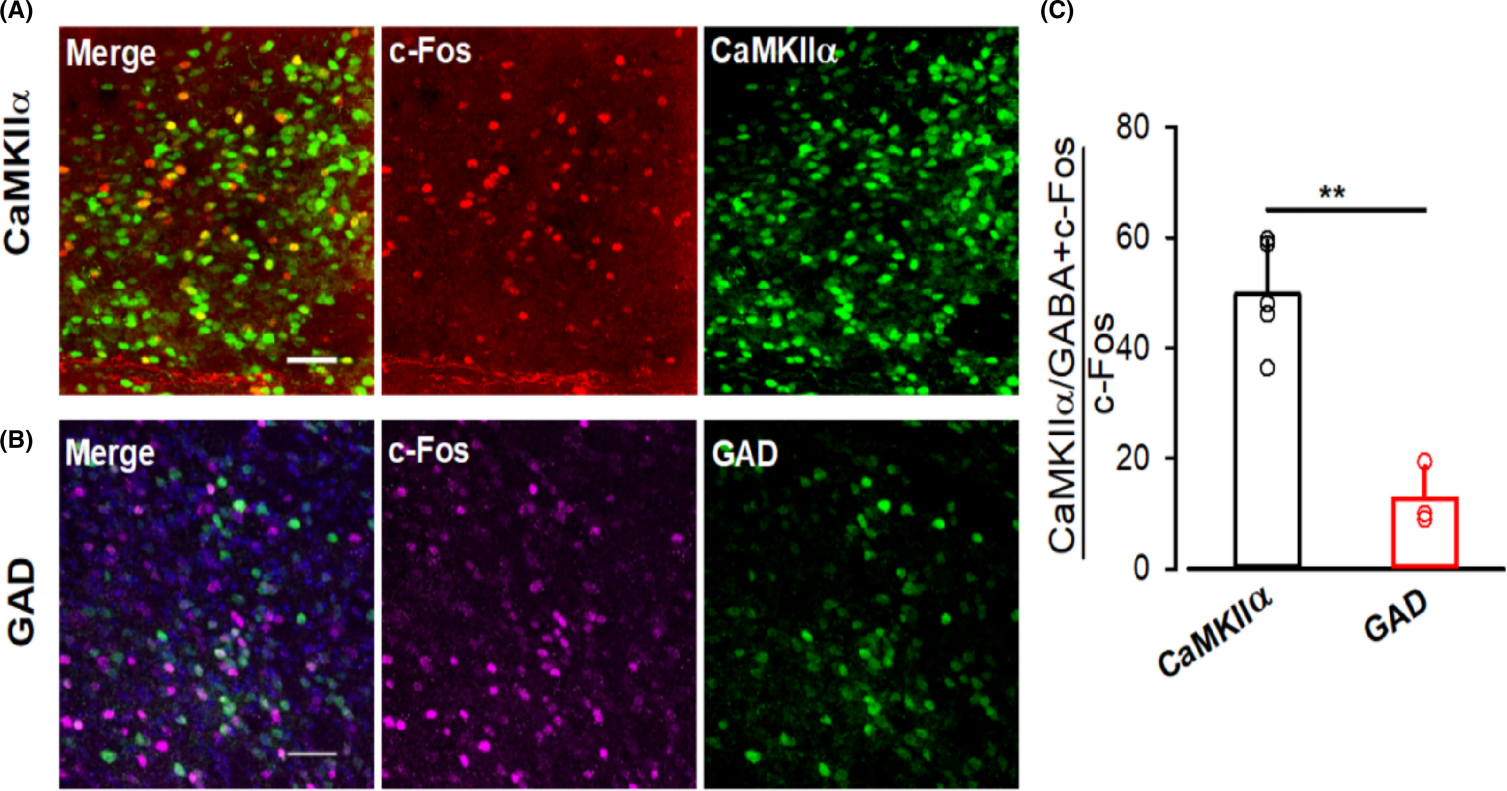
Most neurons activated in the NTS induced by M1 optogenetic activation were excitatory neurons. (A) Representative images of c‐Fos‐positive and CaMKIIα neurons in the NTS after optogenetic activation of M1 with AAV2/9‐CaMKIIα‐ChR2 virus injected into M1 and AAV2/9‐CaMKIIα‐EGFP virus injected into NTS in C57 mice. Scale bar: 50 μm. (B) Representative images of c‐Fos‐positive and GABAergic neurons in the NTS after optogenetic activation of M1 with AAV2/9‐CaMKIIα‐ChR2 virus injected into M1 in GAD67‐GFP mice. Scale bar: 50 μm. (C) More excitatory neurons in the NTS were activated following optogenetic activation of M1 in the CaMKIIα group than in the GAD group (two‐tailed Student's unpaired *t* test, CaMKIIα, *n* = 5; GAD, *n* = 3, *t* = 5.907, **p < 0.01). Data are presented as the mean ± SD, and *n* indicates the number of biologically independent samples (mice) per group.

M1 was confirmed to participate in swallowing function in our previous study and other studies.^26^, ^27^, ^28^ To observe the activity of NTS neurons after M1 activation, the optogenetic activation virus AAV2/9‐CaMKIIα‐ChR2 was injected into M1 L5, followed by blue light stimulation (473 nm) to activate neurons, and c‐Fos‐positive neurons in the NTS were detected after blue light was delivered into M1 (Figure 1D). The results showed that c‐Fos‐positive neurons increased after M1 activation compared to the normal group, or the control group (Figure 1A–C,E; *p* < 0.01). To further explore the types of neurons activated in the NTS, AAV2/9‐CaMKIIα‐ChR2 virus was injected into M1 in C57 mice or GAD67‐GFP mice. To observe excitatory neurons, AAV2/9‐CaMKIIα virus was injected into the NTS, and the types of neurons in the NTS were detected. There was considerable merging of CaMKIIα and c‐Fos neurons in the NTS after optogenetic activation of M1 (Figure 2A,C), but only a small overlap of GAD and c‐Fos neurons was found in the NTS after M1 activation (Figure 2B,C). There was a significant difference between groups (*p* < 0.01). These results indicated that the excitatory neurons in the NTS were mostly activated by M1.

### 3.2 The role of the NTS in swallowing responses induced by the activation of M1

**FIGURE 3:**
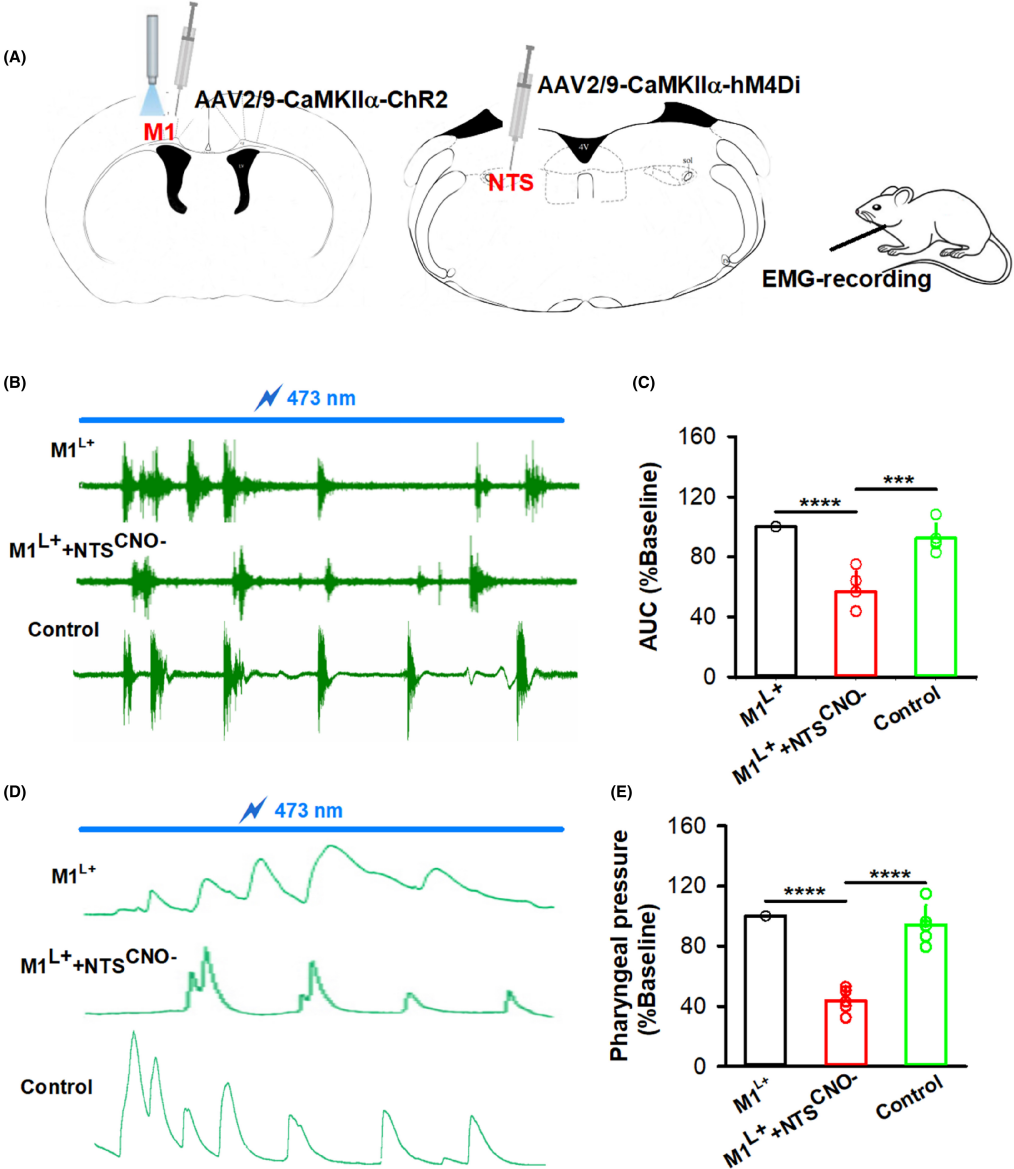
The NTS participated in the swallowing responses induced by optogenetic activation of M1. (A) Diagram showing optogenetic activation of M1 neurons and chemogenetic inhibition of NTS neurons in C57 mice. AAV2/9‐CaMKIIα‐ChR2 virus was injected into M1, and blue light was delivered. AAV2/9‐CaMKIIα‐hM4Di virus was injected into the NTS, and CNO was administered by intraperitoneal injection. Then the EMG response was recorded from the pharyngeal muscle. (B) Representative images of EMG recording for the swallowing response induced by optogenetic activation of M1 in the M1^L+^ and M1^L+^+NTS^CNO−^ groups. The blue line indicates the 473 nm light given to M1 L5. Scale bar: 1 s, 2 mV. (C) The AUC of EMG responses induced by optogenetic activation of M1 was decreased in the M1^L+^+NTS^CNO−^ group compared to the M1^L+^ group. To control for the effect of the hM4Di‐expressing virus, a control group was included (AAV2/9‐CaMKIIα‐ChR2 virus was injected into M1 and blue light was delivered, AAV2/9‐CaMKIIα‐EGFP virus was injected into the NTS and CNO was administered by intraperitoneal injection; one‐way ANOVA, *n* = 5 per group; *F* = 29.49, ****p < 0.0001, M1^L+^ vs. M1^L+^+NTS^CNO−^;***p < 0.001, M1^L+^+NTS^CNO−^ vs. control). (D) Representative images of pharyngeal pressure induced by optogenetic activation of M1 in the M1^L+^, M1^L+^+NTS^CNO−^, and control groups. Scale bar: 1 s, 5 mmHg. (E) The pharyngeal pressure in the M1^L+^+NTS^CNO−^ group was decreased following chemogenetic inhibition of the NTS (one‐way ANOVA, *n* = 5 per group; *F* = 59.36, ****p < 0.0001). Data are presented as the mean ± SD, and *n* indicates the number of biologically independent samples (mice) per group.

Our previous study showed that swallowing function was impaired after NTS inhibition, and EA rescued the expression of c‐Fos‐positive neurons in PSD model mice,^25^ which confirmed the role of the NTS in swallowing function. To further observe the role of the NTS in the regulation of swallowing function by M1, optogenetic activation and chemogenetic inhibition viruses (AAV2/9‐CaMKIIα‐ChR2, AAV2/9‐CaMKIIα‐hM4Di) were injected into the M1 and NTS, respectively (Figure 3A). After chemogenetic inhibition of the NTS, EMG readings of swallowing responses induced by the activation of M1 decreased significantly in the M1^L+^+NTS^CNO−^ group compared to the M1^L+^ group (*p* < 0.0001), while there was no difference in the control group, which was injected with the control virus AAV2/9‐CaMKIIα‐EGFP into the NTS (Figure 3B,C). The same results were observed with pharyngeal pressure detected by a balloon placed over the pharynx during swallowing (Figure 3D,E; *p* < 0.0001).

**FIGURE 4:**
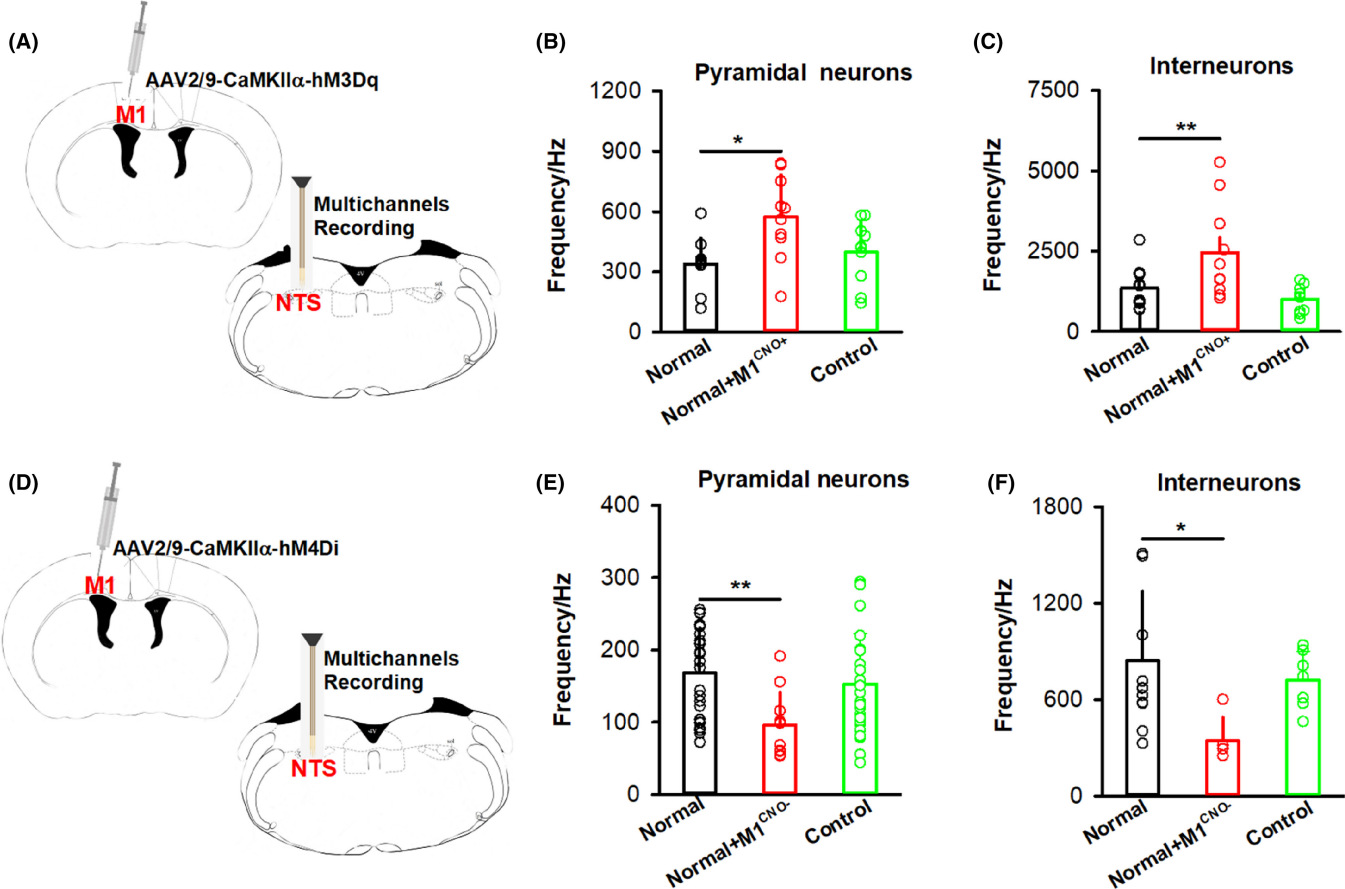
NTS neurons could be regulated by M1. (A) Diagram showing chemogenetic activation of M1 neurons, with AAV2/9‐CaMKIIα‐hM3Dq virus injected into M1 and CNO administered by intraperitoneal injection in C57 mice. A multichannel electrode was implanted into the NTS to record the neuron firing rate of the NTS. (B) Pyramidal neuronal activity in the NTS was significantly increased after chemogenetic activation of M1 neurons in the normal+M1^CNO+^ group compared to the normal group. To control for the effect of the hM3Dq‐expressing virus, a control group was included (AAV2/9‐CaMKIIα‐EGFP virus was injected into M1 and CNO was administered by intraperitoneal injection; one‐way ANOVA, *n* = 8 per group; *F* = 5.286, *p < 0.05, normal vs. normal+M1^CNO+^). A total of 30 units were recorded and 10 units were observed in each group. (C) Interneuron activity in the NTS was significantly increased after chemogenetic activation of M1 neurons in the normal+M1^CNO+^ group compared to the normal group (one‐way ANOVA, *n* = 8 per group; *F* = 6.170, *p < 0.05, normal vs. normal+M1^CNO+^). A total of 30 units were recorded and 10 units were observed in each group. (D) Diagram showing chemogenetic inhibition of M1 neurons, with AAV2/9‐CaMKIIα‐hM4Di virus injected into M1 and CNO administered by intraperitoneal injection. A multichannel electrode was implanted into the NTS to record the neuron firing rate of the NTS. (E) Pyramidal neuronal activity in the NTS was significantly decreased after chemogenetic inhibition of M1 neurons in the normal+M1^CNO−^ group compared to the normal group (one‐way ANOVA, *n* = 5 per group; *F* = 4.863, **p < 0.01, normal vs. normal+M1^CNO−^). A total of 63 units were recorded: 24 units were observed in the normal group, 11 units were observed in the normal+M1^CNO−^ group, and 28 units were observed in the control group. (F) Activity of interneurons in the NTS was significantly decreased after chemogenetic inhibition of M1 neurons in the normal+M1^CNO−^ group compared to the normal group (one‐way ANOVA, *n* = 5 per group; *F* = 3.967, *p < 0.05, normal vs. normal+M1^CNO−^). A total of 24 units were recorded: 12 units were observed in the normal group, 5 units were observed in the normal+M1^CNO−^ group and 7 units were observed in the control group. Data are presented as the mean ± SD, and *n* indicates the number of biologically independent samples (mice) per group.

To further verify the role of the NTS in the regulation of swallowing by M1, the chemogenetic activation virus was injected into M1, and a multichannel electrode was implanted into the NTS to record the neuronal activity of the NTS (Figure 4A). Twenty‐one days later, the firing rates of neurons in the NTS increased after chemogenetic activation of M1 in the normal+M1^CNO+^ group compared to the normal group (*p* < 0.05), whether they were pyramidal neurons or interneurons (Figure 4B,C). No changes were observed in the control group compared to the normal group (*p* > 0.05). Furthermore, the firing rates of both pyramidal neurons and interneurons in the NTS decreased after M1 was chemically inhibited in the normal+M1^CNO−^ group compared to the normal group (Figure 4D–F; *p* < 0.05). In total, the NTS could be regulated by M1 and was involved in the swallowing process modulated by M1.

### 3.3 The NTS was involved in the improvement of swallowing function by EA via the M1 in PSD mice

**FIGURE 5:**
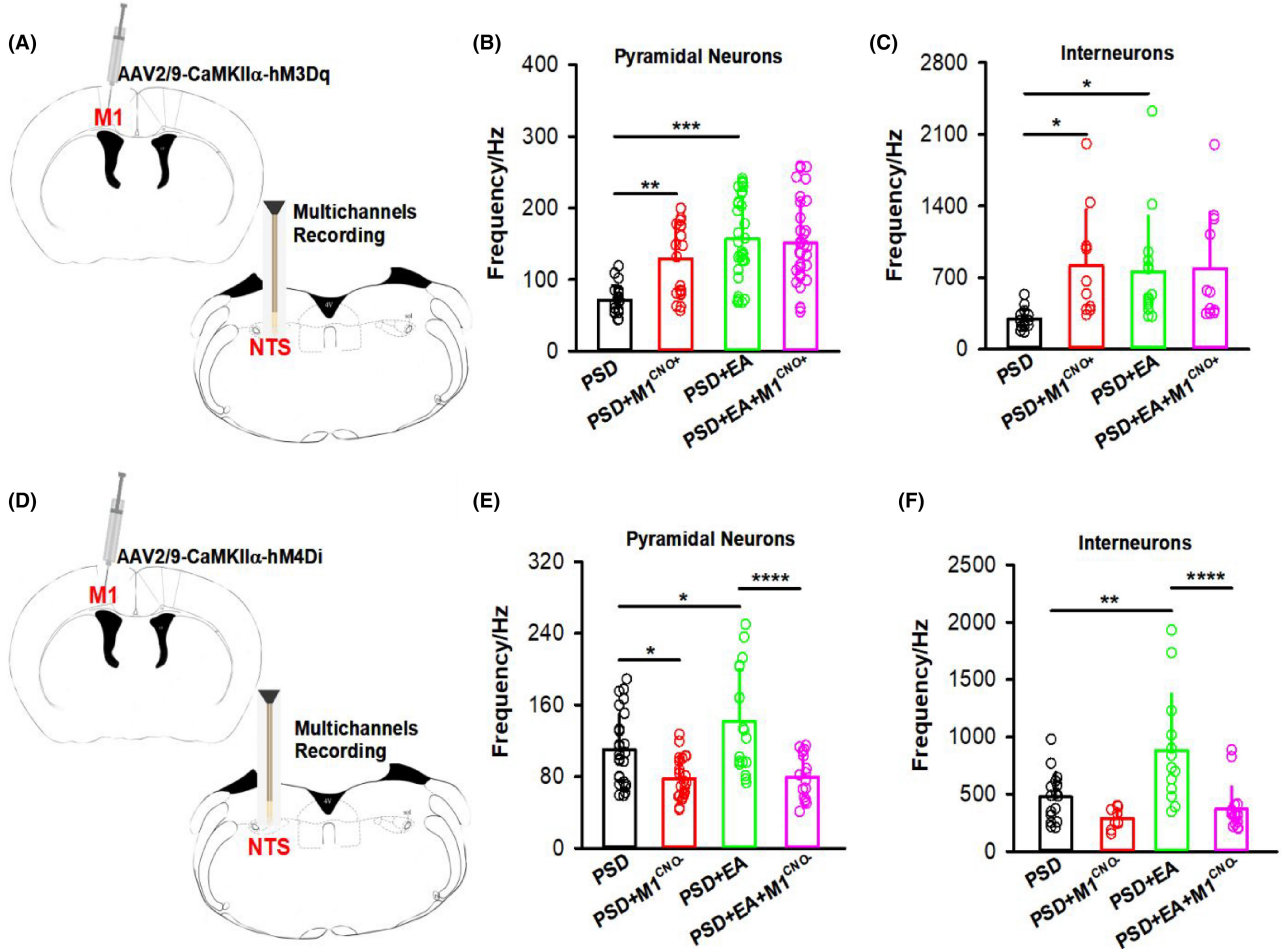
NTS was involved in the regulation of PSD by EA through M1. (A) Diagram showing chemogenetic activation of M1 neurons, with AAV2/9‐CaMKIIα‐hM3Dq virus injected into M1 and CNO administered by intraperitoneal injection in C57 mice. A multichannel electrode was implanted into the NTS to record the neuron firing rate of the NTS. (B) Pyramidal neuronal activity in the NTS was significantly increased after chemogenetic activation of M1 neurons in the PSD + M1^CNO+^ group or EA stimulation in the PSD + EA group compared to the PSD group (PSD, PSD induction; PSD + M1^CNO+^, PSD induction, injection of AAV2/9‐CaMKIIα‐hM3Dq into M1 and CNO intraperitoneal injection; PSD + EA, PSD induction and EA stimulation; PSD + EA + M1^CNO+^, PSD induction, EA stimulation, injection of AAV2/9‐CaMKIIα‐hM3Dq into M1 and CNO intraperitoneal injection; one‐way ANOVA, *n* = 8 per group; *F* = 13.79, **p < 0.01, PSD vs. PSD + M1^CNO+^; ***p < 0.001, PSD vs. PSD + EA). A total of 108 units were recorded: 22 units were observed in the PSD and PSD + M1^CNO+^ groups, and 32 units were observed in the PSD + EA and PSD + EA + M1^CNO+^ groups. (C) Activity of interneurons in the NTS was significantly increased after chemogenetic activation of M1 neurons in the PSD + M1^CNO+^ group or EA stimulation in the PSD + EA group compared to the PSD group (one‐way ANOVA, *n* = 8 per group; *F* = 3.934, *p < 0.05). A total of 50 units were recorded: 15 units were observed in the PSD group, 10 units were observed in the PSD + M1^CNO+^ group, 14 units were observed in the PSD + EA group, and 11 units were observed in the PSD + EA + M1^CNO+^ group. (D) Diagram showing chemogenetic inhibition of M1 neurons, with AAV2/9‐CaMKIIα‐hM4Di virus injected into M1 and CNO administered by intraperitoneal injection. A multichannel electrode was implanted into the NTS to record the neuron firing rate of the NTS. (E) Pyramidal neuronal activity in the NTS was significantly decreased after chemogenetic inhibition of M1 neurons in the PSD + M1^CNO−^ group and increased after EA stimulation in the PSD + EA group compared to the PSD group (PSD, PSD induction; PSD + M1^CNO−^, PSD induction, injection of AAV2/9‐CaMKIIα‐hM4Di into M1 and CNO intraperitoneal injection; PSD + EA, PSD induction and EA stimulation). The pyramidal neuronal activity in the NTS was further decreased after EA stimulation and chemogenetic inhibition of M1 in the PSD + EA + M1^CNO−^ group compared to the PSD + EA group (PSD + EA + M1^CNO−^, PSD induction, EA stimulation, injection of AAV2/9‐CaMKIIα‐hM4Di into M1 and CNO intraperitoneal injection; one‐way ANOVA, *n* = 8 per group; *F* = 11.67, *p < 0.05, PSD vs. PSD + M1^CNO−^, PSD vs. PSD + EA; ****p < 0.0001, PSD + EA vs. PSD + EA + M1^CNO−^). A total of 84 units were recorded: 26 units were observed in the PSD group, 23 units were observed in the PSD + M1^CNO−^ group, 18 units were observed in the PSD + EA group, and 17 units were observed in the PSD + EA + M1^CNO−^ group. (F) Activity of interneurons in the NTS was significantly increased following EA stimulation in the PSD + EA group compared to the PSD group. This neuronal activity was further decreased after EA stimulation and chemogenetic inhibition of M1 in the PSD + EA + M1^CNO−^ group compared to the PSD + EA group (one‐way ANOVA, *n* = 8 per group; *F* = 10.41, **p < 0.01, PSD vs. PSD + EA; ****p < 0.0001, PSD + EA vs. PSD + EA + M1^CNO−^). A total of 55 units were recorded: 16 units were observed in the PSD group, 9 units were observed in the PSD + M1^CNO−^ group, 13 units were observed in PSD + EA and 17 units were observed in the PSD + EA + M1^CNO−^ group. Data are presented as the mean ± SD, and *n* indicates the number of biologically independent samples (mice) per group.

Our previous study showed that M1 participated in the treatment of PSD by EA,^25^ and the NTS was found to be regulated by M1 in our previous section (Figures 3 and 4). To observe whether the NTS participated in the regulation of PSD by EA via M1, a PSD model was established by photothrombotic technique in mice, which was verified in our previous study.^25^ First, the NTS was found to be activated by EA at CV23 in the PSD + EA group compared to the PSD group (Figure S1; *p* < 0.05). Subsequently, chemogenetic activation virus (AAV2/9‐CaMKIIα‐hM3Dq) was injected into M1, followed by multichannel electrode implantation in the NTS to record the neuronal activities of the NTS (Figure 5A). After injection of clozapine (CNO), the firing rates of pyramidal neurons in the NTS increased in the PSD + M1^CNO+^ and the PSD + EA groups compared to the PSD group (*p* < 0.01). However, there were no significant differences after EA and chemogenetic activation of M1 in the PSD + EA + M1^CNO+^ group, compared to the PSD + EA group (Figure 5B; *p* > 0.05). Similar results were found in the interneurons of the NTS (Figure 5C; *p* < 0.05). To further confirm the above observation, a chemogenetic inhibition virus (AAV2/9‐CaMKIIα‐hM4Di) was injected into M1, and the NTS neuronal firing rates were recorded simultaneously (Figure 5D). The results showed that the firing rates of pyramidal neurons in the NTS decreased after inhibiting M1 in the PSD + M1^CNO−^ group compared to the PSD group (*p* < 0.05). EA could increase pyramidal neuronal firing of NTS in the PSD + EA group in comparison with the PSD group (*p* < 0.05). The firing rates of pyramidal neurons in the NTS were further reduced after the inhibition of M1 in the PSD + EA + M1^CNO−^ group compared to the PSD + EA group (Figure 5E; *p* < 0.0001). Similar results were found in the interneurons of the NTS (Figure 5F; *p* < 0.0001).

**FIGURE 6:**
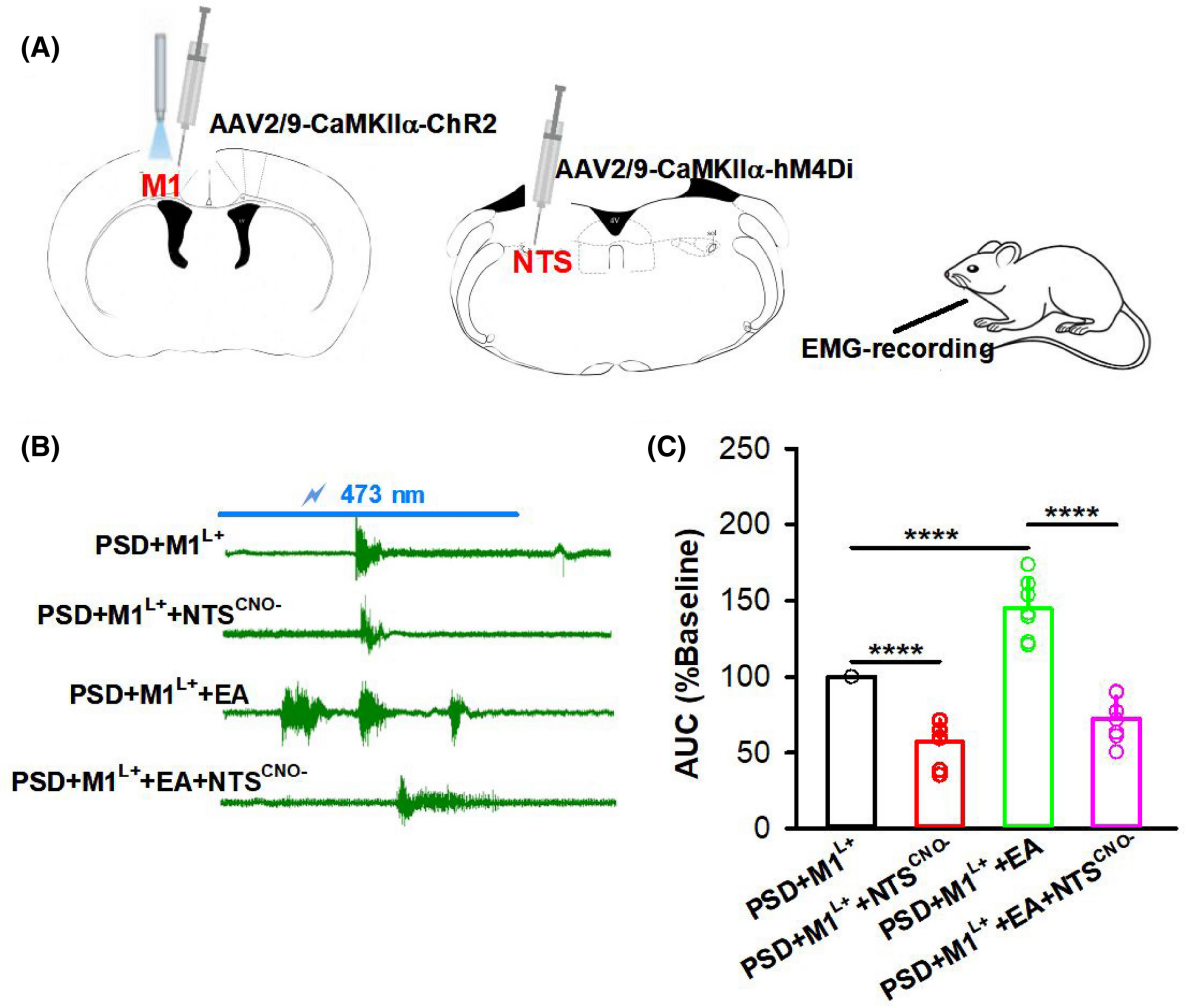
NTS was involved in the regulation of PSD by EA through M1. (A) The experimental scheme showing optogenetic activation of M1, with AAV2/9‐CaMKIIα‐ChR2 virus injected into M1 and 473 nm blue light given to C57 mice. Chemogenetic inhibition of the NTS with AAV2/9‐CaMKIIα‐hM4Di virus injected into the NTS and CNO intraperitoneal injection. Then EMG was recorded from the pharyngeal muscle. (B) Representative images of EMG recording for the swallowing response induced by optogenetic activation of M1 in different groups. (PSD + M1^L+^, PSD induction, AAV2/9‐CaMKIIα‐ChR2 injected into M1 and blue light delivered; PSD + M1^L+^+NTS^CNO−^, PSD induction, AAV2/9‐CaMKIIα‐ChR2 injected into M1 and blue light delivered, injection of AAV2/9‐CaMKIIα‐hM4Di into NTS with CNO intraperitoneal injection; PSD + M1^L+^+EA, PSD induction, AAV2/9‐CaMKIIα‐ChR2 injected into M1 and blue light delivered, EA stimulation; PSD + M1^L+^+EA + NTS^CNO−^, PSD induction, AAV2/9‐CaMKIIα‐ChR2 injected into M1 and blue light delivered, EA stimulation, injection of AAV2/9‐CaMKIIα‐hM4Di into NTS with CNO intraperitoneal injection) Scale bar: 1 s, 0.5 mV. (C) The AUC of EMG responses induced by optogenetic activation of M1 decreased after chemogenetic inhibition of the NTS in the PSD + M1^L+^+NTS^CNO−^ group and increased following EA stimulation in the PSD + M1^L+^+EA group compared to the PSD + M1^L+^ group. The AUC of EMG responses also decreased after EA stimulation and chemogenetic inhibition of the NTS in the PSD + M1^L+^+EA + NTS^CNO−^ group compared to the PSD + M1^L+^+EA group. (one‐way ANOVA, *n* = 7 per group; *F* = 50.92, ****p < 0.0001^−^). Data are presented as the mean ± SD, and *n* indicates the number of biologically independent samples (mice) per group.

To further confirm the role of the NTS in the regulation of PSD by EA via M1, the optogenetic virus (AAV2/9‐CaMKIIα‐ChR2) and the chemogenetic inhibition virus (AAV2/9‐CaMKIIα‐hM4Di) were injected into the M1 and NTS, respectively (Figure 6A). Swallowing responses induced by optogenetic activation of M1 with blue light delivered were recorded by EMG recording. After chemogenetic inhibition of the NTS with CNO intraperitoneal injection, EMG of the swallowing responses induced by the activation of M1 decreased in the PSD + M1^L+^+NTS^CNO−^ group compared to the PSD + M1^L+^ group (*p* < 0.0001). Furthermore, EA at CV23 rescued EMG in the PSD + M1^L+^+EA group (*p* < 0.0001), while this effect was reduced after chemogenetic inhibition of the NTS in the PSD + M1^L+^+EA + NTS^CNO−^ group compared to the PSD + M1^L+^+EA group (Figure 6B,C; *p* < 0.0001). Based on the above results combined, we confirmed that the NTS was involved in the improvement of swallowing function by EA via M1 in PSD mice.

## 4 DISCUSSION

In our study, excitatory neurons in the NTS were activated using immunofluorescence, and optogenetic methods were used to modulate NTS neurons through M1. A PSD mouse model was established using photothrombotic technique, and the function of the NTS regulated by EA via M1 was confirmed. The results showed that the activity levels of both pyramidal neurons and interneurons were changed along with the activation or inhibition of M1, which was involved in the regulation of PSD by EA at CV23. This conclusion could provide neurophysiological evidence for the treatment of PSD by EA.

Immunofluorescence results indicated that more CaMKIIα neurons were activated than GAD neurons in the NTS after activating M1. Previously, excitatory and inhibitory neurons in the NTS were shown to play a key role in the regulation of swallowing function,^29^ including excitatory neurons expressing N‐methyl‐D‐aspartic acid receptors (NMDARs), as well as those expressing α‐amino‐3‐hydroxy‐5‐methyl‐4‐isoxazole‐propionic acid receptors (AMPARs) for excitatory neurons.^30^, ^31^ NMDA‐type receptors have been demonstrated to trigger motor events and form swallowing motor sequences. Regarding inhibitory neurons, gamma‐aminobutyric acid (GABA) is dominant in inhibitory neurons of the NTS, and swallowing is inhibited by injection of GABA agonists into the NTS.^32^, ^33^ Furthermore, other types of neurons are involved in the regulation of swallowing, including serotonergic neurons.^34^ Studies have shown that the swallowing sensory circuit depends on the neurotransmitter 5‐hydroxytryptamine (5HT),^35^ especially given the abundance of the 5HT1a receptor in the NTS. Our previous study also confirmed that 5‐HT1a receptors in the NTS played an important role in initiating swallowing.^36^ Thus, various neurons have been shown to participate in swallowing, resulting in interactions between different neurons during this process.

In our study, both pyramidal neurons and interneurons were regulated by M1 with the same regulatory tendency. The frequency of pyramidal neurons and interneurons increased after M1 activation and decreased after M1 inhibition, which did not correspond to the excitation‐inhibition balance of the NTS, as a previous study showed.^37^ Different types of neurons in the NTS may be regulated by M1 at the same time. In our previous study, pyramidal neurons in M1, traced by a virus injected into the pharyngeal muscles, were found to induce pharyngeal EMG responses.^25^ Our results showed that swallowing responses persisted after inhibition of the NTS, which indicated that there may be another brain region or two involved in the regulation of the M1 or NTS via complex neural regulation. However, the ascending and descending projections of the rostral NTS originate from different neuronal groups, including the forebrain and parabrachial nucleus.^38^ Thus, there may be other types of excitatory neurons participating in swallowing during this regulation through M1. In total, our results on the firing rates of these two types of neurons in the NTS may be influenced by different neurons in one or more regions of the brain.

Recently, our team constructed the most complete matrix of clinical evidence for acupuncture and moxibustion.^39^ Some scholars have noted that the standard acupoints selected for PSD treatment were Lianquan (CV23), Fengchi, and Tiantong, which are the most commonly used among the main acupoints.^40^ CV23 is a commonly used acupoint for the treatment of dysphagia, but its mechanism has been indefinite until now. Researchers believe that CV23 is dominated by sensory fibers of the glossopharyngeal nerve and vagus nerve, and acupuncture at CV23 can stimulate muscles and induce them to contract.^41^ At the same time, the needle sensation caused by acupuncture can enhance sensory input, further increase central excitability, and promote the recovery of swallowing dysfunction.^42^ However, the above studies focused on the peripheral mechanisms of acupuncture. A previous study showed that local field potential and blood flow in M1 could be regulated by EA at CV23 in PSD mice,^27^ identifying a potential therapeutic target for the treatment of PSD. Our recent study revealed the function of the NTS in the treatment of EA in PSD model mice and its indirect role in the motor efferent neural circuit dominated by M1.^25^ In this study, the NTS was directly regulated by M1 activation, which played an important role in the process of EA treatment in PSD mice. However, this effect did not increase after M1 activation and EA stimulation simultaneously, suggesting that the regulatory pathway of EA is consistent with the regulatory effect of M1 on the NTS. Thus, after the activation of M1, the neuronal firing of the NTS reached saturation, and although M1 was reactivated after EA, the neuronal firing in the NTS no longer increased. This effect was further confirmed by chemogenetic inhibition of M1 (Figure 5). Thus, we confirmed that the NTS was involved in the regulation of PSD by EA through M1. Building on the conclusion of our previous study,^25^ this study clarifies the regulatory link between M1 and the NTS.

## 5 LIMITATIONS

Our study focused mainly on the regulation by EA of swallowing motor efferents through M1 on the NTS, without considering the afferents of EA from the periphery to the center, which is a deficiency of our study. Furthermore, it is still being investigated whether other swallowing‐related cortical or subcortical regions participate in the regulation of PSD by EA.

## 6 CONCLUSION

Overall, the central nervous mechanism of EA stimulation at CV23 for dysphagia after stroke was studied based on the regulatory action of the cortex and brainstem, which might provide an innovative theoretical basis for the treatment of PSD in clinical practice. Furthermore, a new treatment that targets distant sites may improve the efficiency of dysphagia treatment.

## AUTHOR CONTRIBUTIONS

Nenggui Xu, Lulu Yao, and Qiuping Ye designed all experiments. Qiuping Ye, Si Yuan, Bing Deng, Yong Dai, and Jiahui Hu performed the experiments. Qiuping Ye and Jia Qiao analyzed the data. Zulin Dou, Nenggui Xu, and Lulu Yao contributed to manuscript preparation. Hongmei Wen and Lulu Yao made significant modifications to the manuscript. Qiuping Ye and Si Yuan wrote the manuscript with the help of all authors.

## FUNDING INFORMATION

The study was supported by the Innovation Team and Talents Cultivation Program of the National Administration of Traditional Chinese Medicine (ZYYCXTD‐C‐202004); the special project of ‘Lingnan Modernization of Traditional Chinese Medicine’ within the 2019 Guangdong Provincial Research and Development Program (2020B1111100008); the Youth Project of the National Natural Science Foundation of China (82202807); China Postdoctoral Science Foundation (2023T160755); and the National Natural Science Foundation of China (81774406).

## CONFLICT OF INTEREST STATEMENT

The authors declare that they have no competing interests. No competing financial or nonfinancial interests from the funders exist.

## Supporting information


Figure S1.

## Data Availability

The data that support the findings of this study are available from the corresponding author upon reasonable request.
