# Supplementary material for: Participation of the nucleus tractus solitarius in the therapeutic effect of electroacupuncture on post‐stroke dysphagia through the primary motor cortex
Source: CNS Neurosci Ther. 2023 Sep 4;30(3):e14442. doi: 10.1111/cns.14442 (PMC10916452; doi:10.1111/cns.14442)
Supplement: Supplementary file 1 — Figure S1. [file CNS-30-e14442-s001.docx]

**Supplementary Figure**


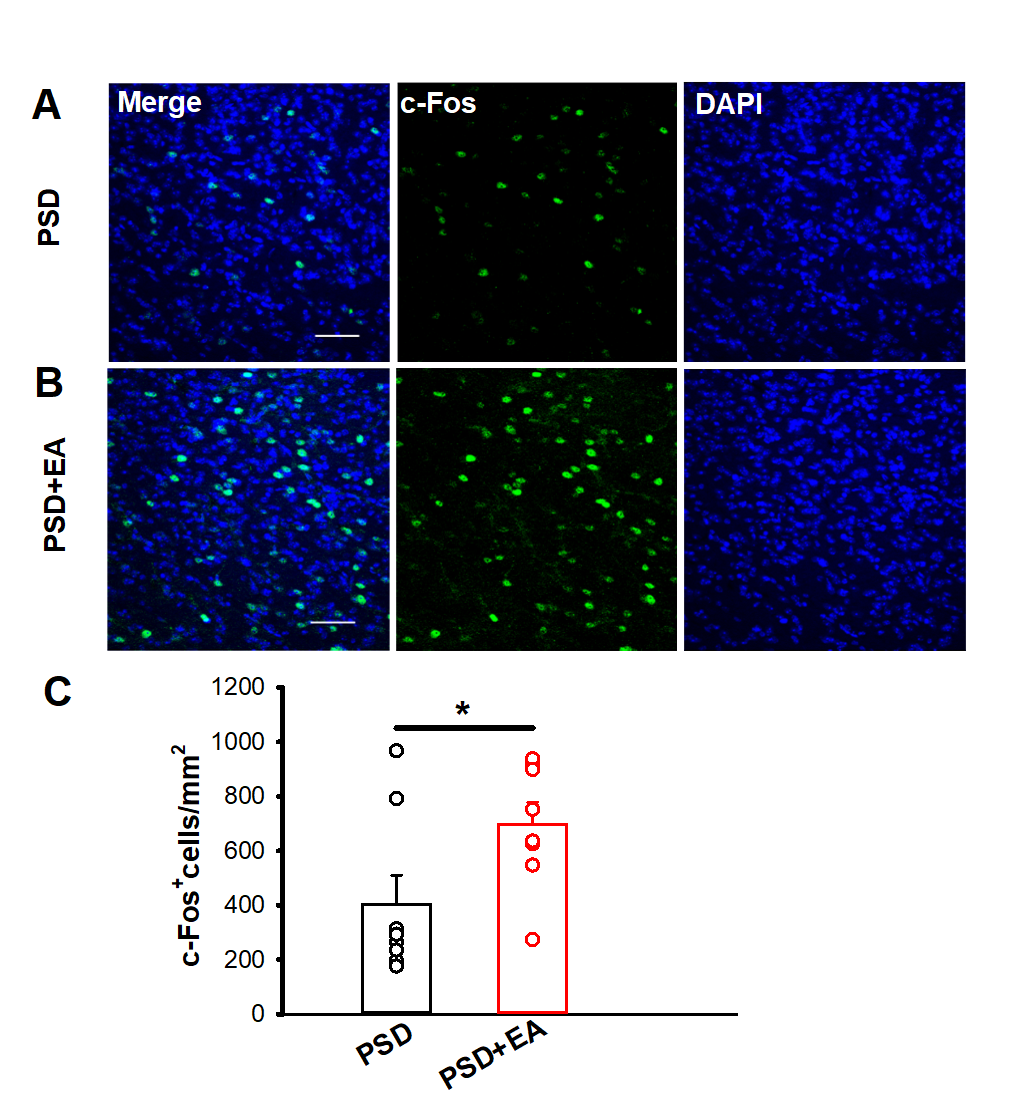


**Sup. Figure** Neurons of the NTS activated by EA at CV23 in PSD mice.

(A-B) Images of c-Fos-positive neurons in the NTS in the PSD (A) and the PSD+EA groups (B) of C57 mice. The left panel shows the merge of c-Fos and DAPI, the middle panel shows c-Fos and the right panel shows DAPI. Scale bar: 50 μm. (C) EA stimulation increased the expression of c-Fos-positive neurons in the NTS in PSD mice. (two-tailed Student’s unpaired t test, n=8 per group, t=3.804 **P<0.05)*. Data are presented as the mean ± SD, and n indicates the number of biologically independent samples (mice) per group.
